# Release Profile of Gentamicin Sulfate from Polylactide-*co*-Polycaprolactone Electrospun Nanofiber Matrices

**DOI:** 10.3390/pharmaceutics11040161

**Published:** 2019-04-03

**Authors:** Silvia Pisani, Rossella Dorati, Enrica Chiesa, Ida Genta, Tiziana Modena, Giovanna Bruni, Pietro Grisoli, Bice Conti

**Affiliations:** 1 Department of Drug Sciences, University of Pavia, Viale Taramelli 12/14, 27100 Pavia, Italy; silvia.pisani01@universitadipavia.it (S.P.); enrica.chiesa01@universitadipavia.it (E.C.); ida.genta@unipv.it (I.G.); tiziana.modena@unipv.it (T.M.); pietro.grisoli@unipv.it (P.G.); bice.conti@unipv.it (B.C.); 2 Polymerix S.r.l., Via Taramelli 24, 27100 Pavia, Italy; 3 Department of Chemistry, University of Pavia, Viale Taramelli 12/14, 27100 Pavia, Italy; giovanna.bruni@unipv.it

**Keywords:** electrospinning, gentamicin sulfate, polylactide-*co*-polycaprolactone, drug release kinetics

## Abstract

The advent and growth of resistance phenomena to antibiotics has reached critical levels, invalidating the action of a majority of antibiotic drugs currently used in the clinical field. Several innovative techniques, such as the nanotechnology, can be applied for creating innovative drug delivery systems designed to modify drug release itself and/or drug administration route; moreover, they have proved suitable for overcoming the phenomenon of antibiotic resistance. Electrospun nanofibers, due to their useful structural properties, are showing promising results as antibiotic release devices for preventing bacteria biofilm formation after surgical operation and for limiting resistance phenomena. In this work gentamicin sulfate (GS) was loaded into polylactide-*co*-polycaprolactone (PLA-PCL) electrospun nanofibers; quantification and in vitro drug release profiles in static and dynamic conditions were investigated; GS kinetic release from nanofibers was studied using mathematical models. A preliminary microbiological test was carried out towards *Staphylococcus aureus* and *Escherichia coli* bacteria.

## 1. Introduction

Electrospinning is a straightforward method of producing polymeric matrices made of ultrafine entangled polymeric fibers with micro- to nano-meter range diameters and controlled surface morphology. The ultrafine fibers are generated by application of a strong electric field on a polymer solution or melt; fibers are stretched and collected on a metallic surface (plate or mandrel). During fiber formation, solvent must evaporate in order to achieve dry and homogeneous fibers. Electrospinning process parameters have been extensively studied and reviewed in the literature in the recent years [1,2].

In these years, the electrospinning technique raised tremendous interest in the pharmaceutical field, as an emerging processing technique of drug delivery systems. Different kinds of drug delivery systems have been investigated to improve the therapeutic effect and to reduce the toxicity of conventional dosage forms. Nanoscale formulations, such as liposomes, polymeric micelles, complexes, and nanofibers, attracted special attention during the last decade. Compared with other formulations, electrospinning affords great flexibility in selecting and combining materials and drugs. Drug molecule encapsulation into electrospun polymer nanofibers, promotes intimate drug to polymer contact, also modifying drug apparent solubility [3,4,5,6,7,8,9,10,11]. As far as electrospun materials are concerned, the ability of reaching high encapsulation efficiencies, simultaneous delivery of diverse drugs, and drug release modification are recognized as significant advantages. Moreover, the electrospinning process itself has notable advantages, such as ease of operation, cost-effectiveness, reproducibility, and easy up-scalability [1,12,13]. The use of electrospun nanofibers as drug carriers is promising in diverse pathologies, such as postoperative local chemotherapy administration wound dressing [14,15].

Several studies can be found in the literature concerning drug release from drug loaded electrospun matrices, either reporting drug release behavior or drug release kinetic evaluation. The latter is an important parameter to be investigated since drug release kinetic gives an indication of drug release mechanism. While a zero-order kinetic with constant release rate is preferred, drug release from polymeric drug delivery systems, such as electrospun nanofibers, in many cases shows initial burst release followed by a more controlled release of the drug over a longer duration.

The behavior depends on the drug dispersion state in the polymer fibers (i.e., molecular dispersion (obtained starting from drug/polymer solution) or solid dispersion (obtained starting from a drug suspension into polymer solution)). Therefore, in order to attain successful encapsulation of a drug into the electrospun nanofibers, the physicochemical properties of polymers, as well as their interaction with the drug molecules, must be precisely considered, as they significantly affect drug-encapsulation efficiency, drug distribution inside the fibers, and, ultimately, drug release from the nanofibers.

Various carrier materials, including natural and synthetic (biodegradable and non-degradable) polymers and a blend of both, were used and are being studied for electrospinning. The drugs investigated for electrospinning belong to different therapeutic classes, such as anticancer drugs, antibiotics and anti-infective, proteins, cardiovascular, DNA, and RNA [9,16,17,18,19,20,21,22,23,24,25]. 

The aim of this work was to prepare electrospun matrices loaded with GS and to in vitro evaluate the drug kinetic profile and its microbiologic activity. In vitro release profiles were attained through conventional static in vitro release test, and through an innovative dynamic bioreactor system that was tested in this work and compared to the conventional in vitro drug release test. The data were elaborated by kinetic release equation models [26]. The GS-loaded electrospun matrices could be used for local controlled drug delivery for treating infected skin and gum or during bone surgery in order to prevent or arrest infection.

Rationale for GS local administration resides in its poor oral bioavailability, and the high occurrence of side effects, such as ototoxicity and toxicity in the kidney, when the drug is administered by intravenous or intramuscular routes, which are the preferential administration routes for Gentamicin. Moreover, topical administration of GS is required with infected burns, excoriations, acne, and impetigo, and in these cases local delivery through a polymer nano-fibrous matrix promoting controlled drug release and prolonged therapeutic effect could be of interest.

## 2. Materials

Copolymer poly-l-lactide-poly-ε-caprolactone (PLA-PCL) 70:30 molar ratio (Resomer LC 703 S – *M*_w_ 160,000 Da), freely soluble in methylene chloride (MC), soluble in *N*,*N*-dimethylformamide (DMF), was obtained from Evonik Industries (Evonik Nutrition and Care GmbH, 64275 Damstadt, Germany). Analytical grade MC and DMF were supplied by Carlo Erba, with no further purification. Gentamicin sulfate, GS (Gentamicin C_1_, C_21_H_43_N_5_O_7_, *M*_w_ 477.6 g/mol, Gentamicin C_2_, C_20_H_41_N_5_O_7_, *M*_w_ 463.6 g/mol, Gentamicin C_1a_, C_19_H_39_N_5_O_7_, *M*_w_ 449.5 g/mol), water solubility 100 mg/mL, insoluble in MC, slightly soluble in DMF (1% *w*/*w*), and ninhydrin (NH), *M*_w_ 178,14 g/mol; purity grade ≥ 95% were from Sigma Aldrich, Milano, Italy. 

## 3. Methods

### 3.1. Preparation of Polymeric Electrospun Fibers Loaded with GS

GS powder (3.3% *w*/*w*) was first dissolved in DMF and then it was added to PLA-PCL solution (20% *w*/*w*, solubilized in MC) and maintained under magnetic stirring at 300 rpm for 2 h, until formation of creaming homogeneous yellowish system. The final PLA-PCL/GS solution, whose composition was 1% *w*/*w* GS and 14% *w*/*w* PLA-PCL, was sonicated to eliminate air bubbles.

The polymer solubility in MC:DMF 70:30 ratio was previously evaluated. The process parameter, set up in a previous work of the research group, were as follows: flow rate 1 mL/h, voltage 20 k/V, needle Gauge 18 [27]. A metallic plate collector covered with aluminum foil was used for avoiding any fiber damage during recovering; syringe distance from collector plate was maintained at 15 cm. The electrospinning process was performed at room temperature (25 °C) and controlled humidity value (~ 50%). Electrospinning time was 30 min in order to achieve nanofiber matrices with suitable GS loading.

### 3.2. UV Quantification of GS

GS quantification was carried out by NH test. Briefly, NH test is a colorimetric assay for quantitative assessment of GS. It is based on the reaction of NH with the primary and secondary gentamicin amino groups leading to the formation of purple compound. The color intensity is directly proportional to gentamicin concentration [28,29]. GS was extracted from electrospun matrices (either the whole matrices or their portions) with the following protocol.

**Figure 1:**
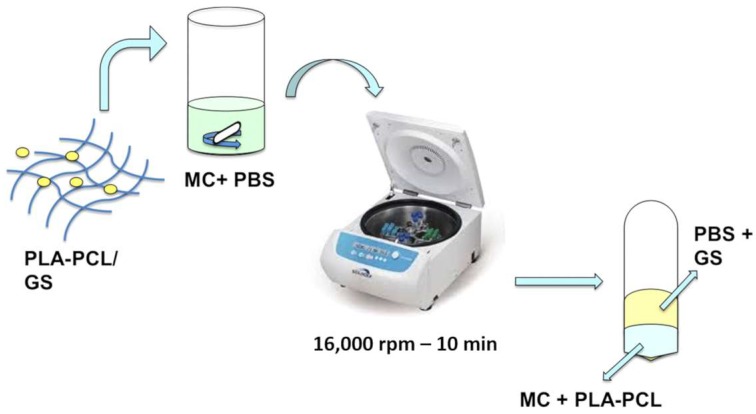
Schematic representation of Gentamicin (GS) extraction from polylactide-co-polycaprolactone (PLA-PCL) electrospun matrices.

For GS recovery, the whole electrospun matrix was solubilized in 3 mL MC and then Phosphate Buffer Saline PBS (4 mL, pH 7.4) were added for extracting GS. The system was maintained under magnetic stirring at 1000 rpm for 1 h to promote GS extraction. The MC/PBS mixture were centrifuged (16,000 rpm, 10 min) in order to separate organic and aqueous phases and recover GS in the aqueous phase. The extraction of GS in the aqueous phase was guarantee by the high affinity of gentamicin for aqueous phase, the poor solubility of PLA-PCL in the aqueous phase, and the partial miscibility organic (MC) and aqueous phases (PBS). After centrifugation, the supernatant containing GS was recovered, and GS quantified by NH assay. A schematic representation of extraction protocol is represented in Figure 1. The extraction protocol was validated and extraction yield was confirmed to be 95 ± 3.0%.

A total of 500 μL of NH solution (5 mg/mL concentration), previously solubilized in PBS was added to 500 μL of supernatant. The final solution was vortexed to ensure complete mixing and subsequently heated to 95 °C for 15 min in order to induce reaction between gentamicin and NH molecules. After 15 min, the solution was stored in water/ice bath for 10 min to stop the reaction. Each sample was analyzed at UV spectrophotometer at 418 nm wavelength, using quartz cuvettes (UV-1601, UV-visible spectrophotometer, Shimadzu, ON, Canada).

PBS solution containing 1% *w*/*w* GS and placebo electrospun matrices were used as controls. All samples underwent the same extraction protocol as GS electrospun matrices before analysis with NH assay.

A six-points calibration curve was obtained with GS standard solutions at concentrations between 0.001 and 0.5 mg/mL. The instrument was calibrated using a blank made of 500 μL of NH solution (0.5% *w*/*v*) added to 500 μL of PBS at pH 7.4.

In order to evaluate drug loading uniformity, all electrospun matrices were cut in a spherical mold. The spherical mold was chosen because when electrospun fibers are collected on a plate collector, they form a circular film that widens as long as fiber deposition is prolonged over time. The reduction and/or prevention in further deposition of fibers could be due to the forming of insulator layer, which depend on electrospun membrane thickness. The mechanism of fiber formation makes that the core of circular film is made of fibers collected from the beginning of the process, while the crown of circular film is made of fiber later collected. Considering no significant differences of drug loading between the core and crown section, it is reasonable to postulate that GS feeding solution was stable during the electrospinning process.

**Figure 2:**
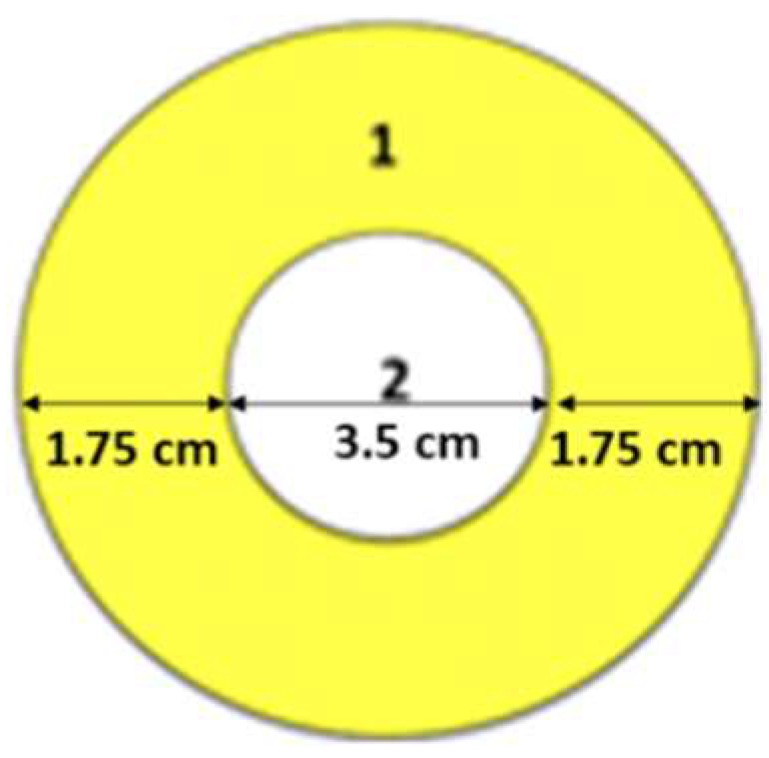
Representation of the two cut concentric portions of electrospun matrices: (1) crown, (2) core.

GS extraction and quantification were performed following the protocol reported above. Each electrospun matrix, whose diameter was 7 ± 0.5 cm, was cut in two concentric parts as shown in Figure 2. Part 1 (identified as crown) having 1.75 cm external circle radius, and part 2 (identified as core) having 3.5 cm diameter. GS was extracted, and in both electrospun matrix molds was quantified. 

Each analysis was performed in triplicate, and average values and standard deviations (sd) were calculated. The reference for GS content percentage determination was the theoretical amount of GS loaded in the matrix calculated as follows:

GS content (%) = mg GS in matrix/mg GS in the electrospun polymer solution × 100

### 3.3. Morphology Characterization by Scanning Electron Microscopy (SEM)

SEM analysis was carried out on placebo and GS-loaded electrospun matrices in order to characterize their morphology in term of nanofiber size, shape, and orientation. Zeiss EVO MA10 apparatus (Carl Zeiss, Oberkochen, Germany) was used with 5000× magnification. Nanofiber size was determined by digital elaboration of SEM images with ImageJ software (National Institutes of Health (NIH) open source image processing program). 

### 3.4. In Vitro GS Release Test

In vitro drug release tests were performed at 37 °C, both in static and dynamic conditions, in order to evaluate how medium flow rate and its direction affect drug release profile. The tests were carried out on both the whole electrospun matrices and on portions of the electrospun matrices that were cut as explained above.

#### 3.4.1. In Vitro GS Release Test in Static Conditions

Each sample was placed in Erlenmeyer flasks and soaked in 10 mL of PBS (pH 7.4) at 37 °C in a thermostatic chamber. Medium was withdrawn (1 mL) at scheduled times up to GS complete release; PBS was restored with fresh buffer at each withdrawal. 

#### 3.4.2. In Vitro GS Release Test in Dynamic Conditions

**Figure 3:**
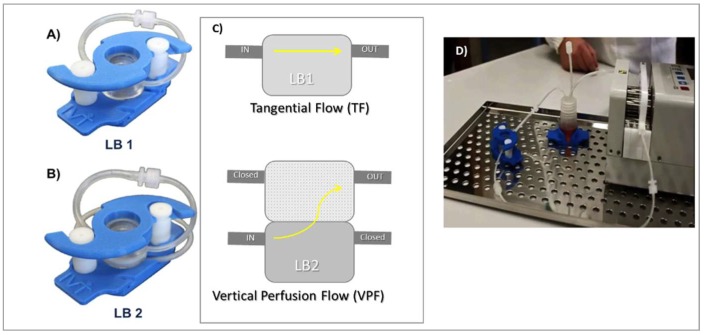
(**A**) Live Box1 (from IVTech website); (**B**) Live Box 2 (from IVTech website); (**C**) IVTech live Box 1 and scheme and flow configuration: Tangential Flow (TF) and Vertical Perfusion Flow (VPF); and (**D**) IVTech system equipped with pump and tubing systems.

In vitro release tests in dynamic conditions were performed using IVTech Livebox1 (LB1) and Livebox2 (LB2) combined with a peristaltic pump (IPC4 - ISMATEC), as shown in Figure 3. IVTech LiveBox 1 is a bioreactor made of a transparent silicon cell with a capacity of 1.5 mL and tangential flow perfusion of the incubation medium, (Figure 3A). The cell is delimitated both on the upper side and on the lower side by little glass disks (Ø 20 mm), and the locking system guarantees closure and endurance during the test. IVTech LiveBox2 (LB2) has the same structural characteristics of LB1 but with vertical perfusion flow configuration (down–top), (Figure 3B). 

The PBS buffer (10 mL, pH 7.4) was continuously recirculated from the reservoir vial (see Figure 3C) at two different flow rates: 0.3 mL/min and 0.6 mL/min maintaining temperature at 37 °C. IVTech LiveBox has peculiar characteristics; the sample is in contact with a small volume of incubation medium simulating the most physiologic condition of implantation into the human body. The flow through the cell creates dynamic conditions; the flow rate can be selected in the range 0.1–0.4 mL/min for LiveBox 1 (TF), and up to 0.5 mL/min in apical or basal compartments for LiveBox 2 (VPF). Flow direction can be either tangential or vertical perfusion.

In vitro release test was performed on small portions of electrospun matrix sections (1 and 2) because of the limited IVTech LiveBox chamber size. The final data were expressed as averages of the two small parts. All data were normalized against their weight in order to make them comparable with data obtained for whole matrices.

#### 3.4.3. Kinetic Release Equations

Most common kinetic profiles considered to describe drug release are zero-order, first-order, Higuchi model, or Korsmeyer-Peppas [27,28,29,30,31]. The in vitro release data were elaborated using the following kinetic release equation models:

Zero-order model

*Q*_t_ = Q_0_ + K_0_*t* (1)

where Q_t_ is the amount of drug determined in the incubation medium at the fixed times, *t* is the time spans, and K_0_ is the zero-order release constant expressed in units of concentration/time. The model fits drug release from dosage forms that do not disaggregate and where drug release rate is independent of its concentration. The model is used to describe drug dissolution from several types of modified release pharmaceutical dosage forms.

First-order release model

Log *Q*_t_ = LogQ_0_ − k_1_ t/2.303 (2)

where *Q*_t_ and Q_0_ are the amounts of drug released at time *t* and at time zero respectively, K_1_ is the first order release constant, and *t* is time. Drug release rate depends on its concentration; this model can be used to describe drug dissolution in pharmaceutical dosage forms containing water-soluble drugs in porous matrices.

Higuchi model

*Q* = K_H_*t*^1/2^ (3)

Equation (3) represent the simplified Higuchi model where *Q* is the amount of drug released in time *t* and K_H_ is the Higuchi dissolution constant. The amount of drug released in the fixed time spans, represented as function of the square root of time, fits a straight line.

The Higuchi model is suitable as model for drug release from thin films, containing finely dispersed drugs into perfect sink conditions; this model suggests that drug release is by diffusion, and it takes into account matrix porosity and tortuosity. The premise of the kinetic model is that perfect sink conditions are attained in the release environment.

Korsmeyer-Peppas model

*M*_t_/*M* = k *t^n^* (4)

where *M*_t_ and *M* are the amount of drug at time *t* and loaded into the drug delivery system respectively, *k* is the kinetic constant related to the delivery system and encapsulated substance properties. *n* is release exponent and depends on the type of transport, geometry, and polydispersity of solute; it illustrates the solute transport mechanism as follows: (i) *n* < 0.5 corresponds to a pseudo-Fickian behavior of diffusion; (ii) *n* = 0.5 suggests Fickian behaviour; (iii) 0.5 < *n* < 1 indicates an anomalous diffusion; (iv) *n* = 1 shows non-Fickian diffusion. The amount of drug released in the fixed time spans is represented on a log–log basis.

### 3.5. In Vitro Degradation Study: Mass Loss Analysis

Degradation study, evaluated on gravimetric basis: mass loss % (ML %) in the dissolution medium, was monitored on a parallel set of samples along all in vitro release test times, and it was determined with the following protocol. The initial mass of the matrix (M_0_) was determined by weighing the lyophilized samples with an analytical balance (Mettler Toledo, AG245 mod, Milano, Italy) before being subjected to in vitro degradation tests (time zero).

The samples were incubated in PBS pH 7.4 at 37 °C, the same conditions as for the in vitro release test were followed. Each sample was taken from the buffer, at the sampling times fixed for the degradation test (3, 5, 14, 21, and 28 days), it was washed with distilled water to remove the water-soluble oligomers that can form due to copolymer degradation, and lyophilized (Lio 5P) at −48 °C at 0.4 mbar for 12 h to eliminate all water traces. The lyophilized samples were subsequently weighed (*M_x_*). Mass loss was calculated using the following equation:

*Mass Loss* (%) = *M*_0_ − *Mx*/*M*_0_ × 100

### 3.6. Antibacterial Activity Measurements

The antimicrobial activity of GS-loaded electrospun matrices (EL-GS) was evaluated against the following reference bacterial strains: *Staphylococcus aureus* ATCC 6538 and *Escherichia coli* ATCC 10356. Before testing, bacteria were grown overnight in Tryptone Soya Broth (TSA, Oxoid, Basingstoke, UK) at 37 °C. The cultures were centrifuged at 224 g for 20 min, in order to separate the microorganisms from the culture broth, and then washed with purified water. Washed cells were further suspended in Dulbecco’s PBS (phosphate buffered saline, Sigma-Aldrich, Milan, Italy) and optical density (OD) was adjusted to 0.3, corresponding approximately to 1 × 10^8^ colony forming units (CFU)/mL at 650 nm wavelength.

The antimicrobial activity was evaluated in the presence of the EL-GS, or a placebo to be used as a control. Viable microbial counts were evaluated after contact for scheduled time with EL-GS and with placebo samples (electrospun matrices without GS); bacterial colonies were enumerated in TSA after incubation at 37 °C for 24 h. The microbicidal effect (ME value) was calculated for each test organisms and contact times according to the following Equation [32]:

*ME* = log *N*c − log *N*d (5)

where *N*c is the number of CFU of the control microbial suspension and *N*d is the number of CFU of the microbial suspension in presence of patches.

## 4. Results

### 4.1. Preparation of Polymeric Electrospun Fibers Loaded with GS

The GS-loaded electrospun matrices, obtained after 30 min electrospinning, were round matrices whose diameter was 7 ± 0.5 cm and with average weight value of 58 ± 2.9 mg, as determined on six replications obtained in the same experimental conditions reported in the method section. 

### 4.2. UV Quantification of GS

**Figure 4:**
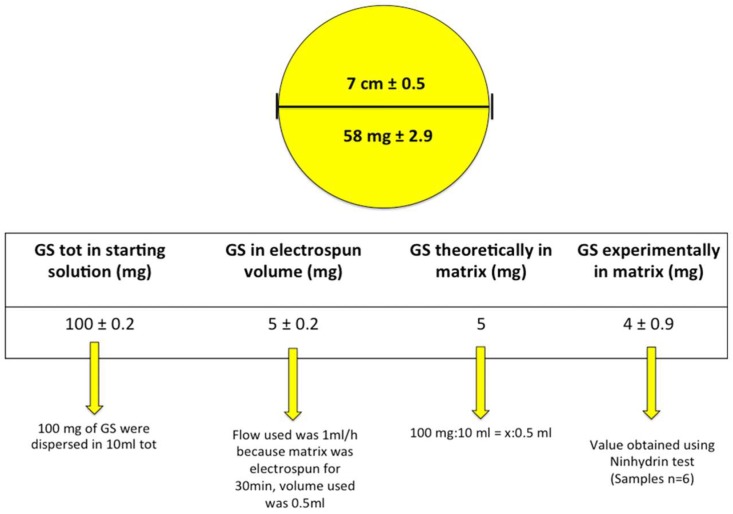
GS quantification in electrospun PLA-PCL matrices. The figure refers to GS amount in the whole electrospun matrix.

The results of GS quantification in the whole electrospun matrices are reported in Figure 4.

**Figure 5:**
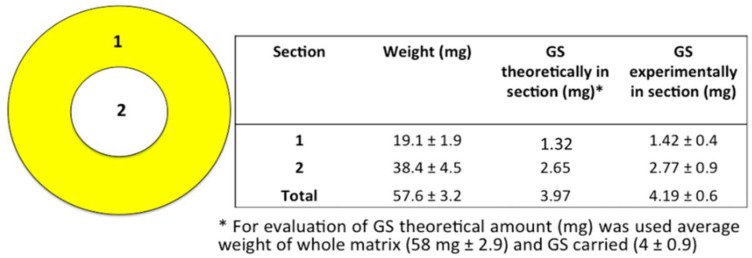
GS quantification in matrices portions.

The amount of GS experimentally determined in the electrospun matrices, expressed as relative mass ratio of the loaded drug to the fiber, was 6.8 *w*/*w*%, and corresponded to the GS theoretically calculated amount. Slight loss of GS micrograms was due to the cleaning step during the electrospinning process. The results of GS determination in the matrix’s portions are reported in Figure 5. GS content result was uniform compared to weight, in the two sections considered. This confirmed GS was uniformly dispersed in the polymer solution during the electrospinning process.

### 4.3. Morphologic Characterization

**Figure 6:**
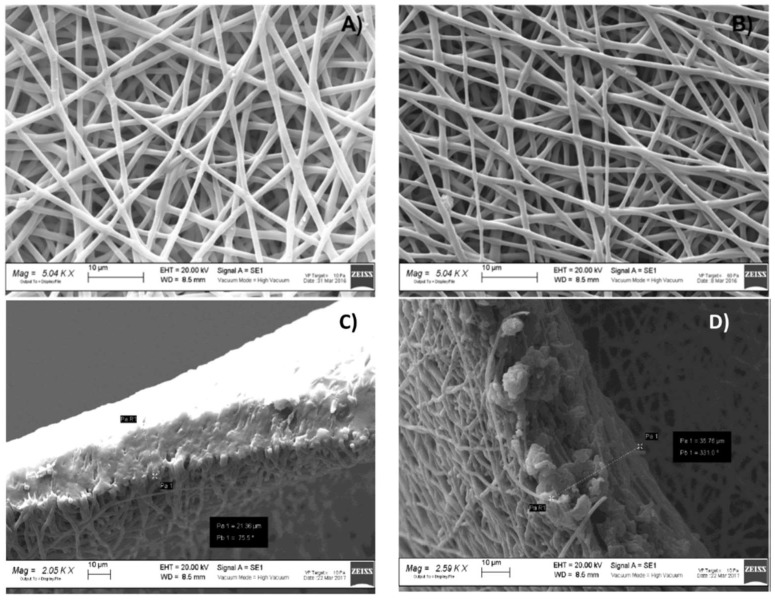
Scanning Electron Microscopy (SEM) images of (**A**) PLA-PCL electrospun matrices (magnification 5.04 KX); (**B**) PLA-PCL/GS electrospun matrices (magnification 5.04 KX); (**C**) orthogonal section of electrospun matrices portion 1 (external edge) (magnification 2.05 KX); and (**D**) orthogonal section of electrospun matrix portion 2 (core) (magnification 2.59 KX).

Data obtained by SEM analysis (Figure 5) showed that fibers morphologies were unchanged after GS addition in polymeric solution. The fibers were in a 700–800 nm range dimension with a smooth surface. No evidence of GS crystals, either on nanofibers surface (Figure 6A,B) or in the electrospun matrices (Figure 6C,D), was highlighted by SEM analysis. Electrospun matrix thickness, as measured by SEM analysis on six replications, resulted to be significantly greater for core portion, 41.81 ± 0.21 µm, with respect to external portion that was 28.61 ± 1.02 µm. The result was due to the electrospinning process on a plane collector and without any restriction, which led to the accumulation of fibers, starting from the collector central part and enlarging the radius as long as conductivity changed as a function of fiber deposition on the collector. As 30 min was fixed as the electrospinning time, this caused the fabrication of matrices with different thickness, decreasing from core to edges. Fibers entanglement, size, and shape did not change in the two different portions analyzed, while fiber density was greater in the electrospun matrices’ central portions. 

### 4.4. GS In Vitro Release in Static and Dynamic Conditions

**Figure 7:**
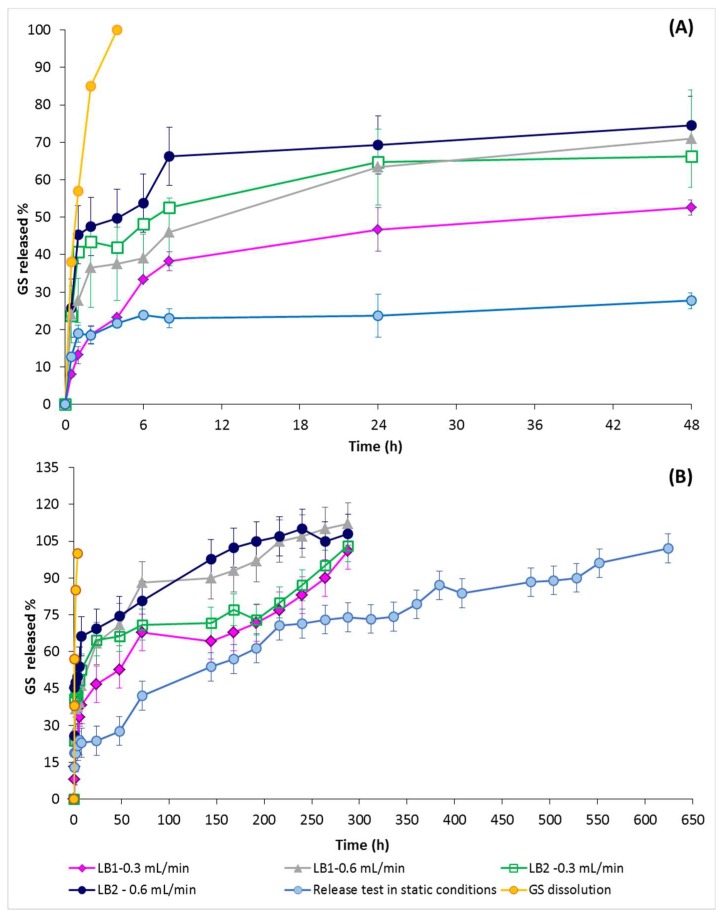
GS in vitro release profiles as tested in static and dynamic conditions: (**A**) GS release in the first 48 h test; and (**B**) in vitro release profiles at GS release completion.

GS in vitro release profiles obtained either in static or dynamic condition tests were collected in Figure 7, showing that electrospun nanofibers significantly slowed down GS release with respect to drug pristine dissolution profile (Figure 7, orange curve). 

GS release from the electrospun matrices, tested in static conditions, was completed in 624 h (26 days). No differences were highlighted between testing the whole electrospun matrices or a portion of them (data not reported), corroborating that GS was uniformly distributed in the electrospun matrix. Moreover, since GS solubility in water is 100 mg/mL, the in vitro release test on the electrospun matrices was conducted always in sink conditions. For these reasons, the different matrix thicknesses did not play a significant role in GS release. GS release profiles in dynamic conditions using IVTech bioreactors (LB1 and LB2) were reported in Figure 7.

Drug release in dynamic conditions, as expected, was significantly faster with respect to drug release rate in static conditions. In the first 24 h of testing, drug release was faster with LB2 bioreactor, which means that orthogonal flow, independently from flow rate, promoted drug release. However, drug release profiles after the first 24 h showed that the higher flow rate (0.6 mL/min) significantly sped up drug release and the higher flow rates (0.6mL/min) corresponded to significantly faster drug release with both types of flows tested (tangential for orthogonal flow). Moreover, flow direction did not seem to significantly affect the GS release profile when lower the flow rate was applied (0.3 mL/min); however, in this case, complete GS release was achieved in 288 h, both using LB1 and LB2 bioreactors.

Drug burst release (drug released in the first 8 h test) was highlighted in all the in vitro release conditions tested, and it increased by incrementing dissolution medium flow rate and changing from tangential to vertical perfusion flow direction. The results are consistent with GS high water solubility that makes drug release highly sensitive to environmental conditions.

The in vitro release data were elaborated by kinetic release equation models and results are reported in Table 1 and Table 2. 

Electrospun matrices showed drug release was regulated from the Higuchi kinetic model, both in dynamic and static conditions. The Korsmeyer-Peppas slope exponent (*n*) was between 0.1 and 0.25, which confirms that the pseudo-Fickian diffusional mechanism controlled the GS release from electrospun nanofibers in all the conditions analyzed.

### 4.5. In Vitro Degradation Test: Mass Loss Analysis

**Figure 8:**
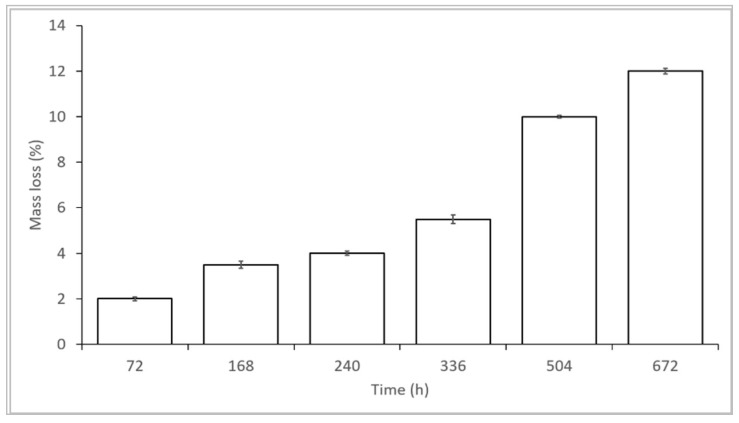
GS-loaded electrospun matrices’ mass loss percentage in simulated physiologic conditions (PBS, pH 7.4).

The mass loss results in the 28 days, corresponding to in vitro release test, are reported in Figure 8. They show that matrices’ mass loss reached 12% after 28 days incubation in simulated physiologic conditions. The results were consistent with data discussed by the authors in a previously published work [27] on PLA*-P*CL placebo electrospun matrices, showing GS-loaded PLA-PCL electrospun matrices are stable in the 28 days incubation in the simulated physiologic conditions tested.

### 4.6. Antibacterial Activity Measurements

The results of antibacterial activity measurements are reported in Table 3, they show the antimicrobial activity obtained against both *S. aureus* and *E. coli*. In particular, the values of ME were sufficiently high to reach a full antimicrobial effect at the concentrations investigated, comparable to the action of a disinfectant (ME ≥ 4) [32,33].

CEN EN 13697 reports ME should be tested after 15 min of contact for disinfectant antibacterial activity determination. Here an antibiotic drug embedded in a polymer matrix was tested; therefore, in a different situation, slightly different time parameters were set (1 h) in order to consider GS release rate from the electrospun matrices. 

The antimicrobial effect, being significant after 1 h of contact, increased with time consistently with GS release from the electrospun matrices. Placebo polymeric electrospun matrices show null antimicrobial effect against both microbial strains tested. Considering that in 48 hours the EL-GS released about 27.7% GS in the same conditions (see Figure 7B, GS release in static conditions), it can be hypothesized that the antibacterial activity could prolong with time. 

## 5. Discussion

In this work GS was solubilized in DMF, and the mixture solvent system (MC:DMF 70:30) aided the physical stabilization of GS in the polymer solution during the electrospinning process. GS concentration in the final solvent mixture, hence the amount of GS loaded into the electrospun matrices, was strictly dependent on GS solubility in the solvents used. In this experimental condition, uniform distribution of GS inside the electrospun mats was obtained, permitting an accurate dosage of GS, and no crystals were observed by SEM analysis of the electrospun matrices. 

GS-loaded electrospun matrices were recently investigated by Coimbra and coll [24], demonstrating the interest in GS local delivery by polymer electrospun matrices. The authors loaded GS into electrospun matrices, starting from a suspension or an emulsion; in all cases, they highlighted similar drug release profiles with great burst release.

The results of the present experimental work highlighted that GS release rate from the electrospun matrices was slowed down with respect to in vitro dissolution of GS powder. Attention was focused on the different in vitro release tests performed, in static conditions and dynamic conditions, and results highlighted that medium flow rate significantly affects drug release rate. This could be explained by a higher differential between drug concentration at the stationary state surrounding the matrices surface and bulk drug concentration, when in vitro release test was carried out in dynamic conditions. The hypothesis was confirmed by high evidence of the phenomenon at the beginning of in vitro release test. Moreover, GS release followed a Higuchi kinetic model, typical of diffusion through porous thin films. The result is consistent with the low values of polymer mass loss obtained, demonstrating that degradation of the PLA-PCL electrospun matrix was not involved in GS release electrospun matrices. 

## 6. Conclusions

The results indicate that there is a good and suitable chance for the future use of electrospun nanofibers as carriers for antibiotics. The electrospun matrices could be applied on severe burns, in order to prevent infections after its implantation into gingival cavities for local infection treatment, or after tooth explant. The advantage is the ability to reach high antibiotic concentrations at the site of action, in the meantime avoiding high system concentrations, thus reducing the drug side effects. Moreover, the prolonged effect of antibiotic at the site of action can reduce administration frequency and improve patient compliance. As far as GS is concerned, further studies should be conducted to ascertain how to reduce GS burst release from the electrospun matrices, meanwhile rendering drug release more independent from environmental conditions. 

## Figures and Tables

**Table 1:** Kinetic model elaboration of GS release from EL matrices incubated in static conditions in (pH 7.4) at 37 °C.

Models	Intercept	Slope	*R* ^2^
**Zero-order**	0.02	1	0.94
**First-order**	−1.76	1.2 × 10^−2^	0.93
**Higuchi**	0.01	2.5 × 10^−3^	0.97
**Korsmeyer-Peppas**	−1.88	2.3 × 10^−1^	0.77

**Table 2:** Kinetic model elaboration of GS release from electrospun matrices incubated in dynamic conditions in PBS (pH 7.4) at 37 °C.

	TF-LB1 Bioreactor						VPF – LB2 Bioreactor					
Flow Rate (mL/min)	0.3			0.6			0.3			0.6		
	Intercept	Slope	*R* ^2^	Intercept	Slope	*R* ^2^	Intercept	Slope	*R* ^2^	Intercept	Slope	*R* ^2^
**Zero order**	0.01	0.0001	0.77	0.01	0.0003	0.85	0.02	−7 × 10^−5^	0.77	0.03	−7 × 10^−5^	0.85
**First order**	−2.03	0.003	0.63	−1.51	0.003	0.78	−1.59	1.4 × 10^−3^	0.82	−1.59	−1.3 × 10^−3^	0.84
**Higuchi**	0.007	0.0015	0.90	0.023	0.004	0.93	0.03	−1.1 × 10^−3^	0.98	0.03	−1.1 × 10^−3^	0.98
**Korsmeyer-Peppas**	−2.15	0.25	0.87	−1.56	0.17	0.83	−1.55	−8.86 × 10^−2^	0.82	−15,505	−9 × 10^−2^	0.96

**Table 3:** Antibacterial activity of EL-GS evaluated against *S. aureus* and *E. coli*.

Sample Code	Contact Time (h)	Microbicide Effect (ME) vs. *S. aureus*	Microbicide Effet (ME) vs. *E. coli*
EL-GS *	1.0	3.79	5.86
EL-GS	6.0	7.21	8.04
EL-GS	24	10.15	10.37
EL-GS	48	10.48	10.69
EL	1.0	1.0	1.0
EL **	6.0	1.0	1.0
EL	24	0	0
EL	48	0	0

* EL-GS: Electrospun matrices loaded with GS; ** EL: placebo electrospun matrices.
